# Does Sexual Orientation Influence Trajectories of Change in Health? A 20-Year Follow-Up Study

**DOI:** 10.1093/geroni/igaa057.1668

**Published:** 2020-12-16

**Authors:** Christi Nelson, Ross Andel

**Affiliations:** University of South Florida, Tampa, Florida, United States

## Abstract

We examined the differences in physical health outcomes over a 20-year period between lesbian, gay, and bisexual (LGB) and heterosexual middle-aged and older adults. We also examined whether the associations were moderated by social support and affect. The analytical sample included 168 LGB adults and 336 propensity-matched heterosexual adults from the Midlife in the United States (MIDUS) study, ranging in age from 25 to 74 years (mean age=42.83) at baseline. Using negative binomial generalized estimating equations and mixed-effects analyses, data from three waves of MIDUS, spanning approximately 20 years from 1995 to 2014, were used to examine the associations between sexual orientation and the health outcomes (number of chronic conditions and functional limitations). Social support and affect were added to the models to test for moderation. The results found that LGB participants reported one more chronic condition at baseline and scored significantly higher for functional limitations. However, LGB participants increased less over time for number of chronic conditions than heterosexual participants, and there were no significant differences in terms of changes in functional limitation over time. Positive affect reduced the strength of the relationship between sexual orientation and functional limitations for LGB participants. No other moderating effects were significant. The results of this study suggest that LGB individuals may become resilient to the negative health effects of minority stressors over time. Interventions should focus on improving the health of LGB individuals when they are younger and more at risk of negative health outcomes.

